# Glucocentric Drugs in Cardiovascular Disease Protection and Heart Failure

**DOI:** 10.14797/mdcvj.1155

**Published:** 2022-12-06

**Authors:** Khawaja M. Talha, Gregg C. Fonarow, Salim S. Virani, Javed Butler

**Affiliations:** 1 Department of Medicine, University of Mississippi Medical Center, Jackson, Mississippi, US; 2 Ahmanson-UCLA Cardiomyopathy Center, Division of Cardiology, University of California Los Angeles, Los Angeles, California, US; 3 Section of Cardiovascular Research, Baylor College of Medicine, Houston, Texas, US; 4 Section of Cardiology, Michael E. DeBakey Veterans Affairs Medical Center, Houston, Texas, US; 5 Baylor Scott & White Research Institute, Baylor University Medical Center, Dallas, Texas, US

**Keywords:** sodium-glucose cotransporter-2 inhibitor, glucagon-like peptide 1 receptor agonist, heart failure, cardiovascular disease, prevention

## Abstract

Evidence for cardiovascular outcomes with older-generation antihyperglycemic drugs in the management of type 2 diabetes is based on aggregated data from prior randomized controlled trials and observational studies that were not focused on prespecified cardiovascular end points. Newer antihyperglycemic medications have undergone a rigorous evaluation of cardiovascular outcomes through randomized controlled trials since the US Food and Drug Administration imposed a mandatory requirement for all glucose-lowering drugs in 2008. The three classes of drugs that have been most extensively studied are dipeptidyl peptidase 4 inhibitors, glucagon-like peptide 1 receptor agonists, and sodium-glucose cotransporter-2 (SGLT2) inhibitors, the latter two reporting significant reductions in adverse cardiovascular outcomes independent of their glycemic effect. Remarkably, it was the evidence from SGLT2 inhibitors cardiovascular outcome trials that prompted further evaluation of the drug class in patients with heart failure irrespective of their diabetes status, demonstrating a broader cardiometabolic effect of these drugs. In this review, we assess the evidence for cardiovascular outcomes with common older- and newer-generation glucose-lowering drugs in the management of type 2 diabetes. We also discuss emerging glucose-lowering drugs with novel metabolic targets that influence the risk of adverse cardiovascular events and expand on the role of these drugs beyond the management of type 2 diabetes.

## Introduction

The diagnosis of type 2 diabetes mellitus (T2DM) is almost synonymous with an impending cardiovascular (CV) event, even with optimal glycemic control. It is associated with a 4-fold increase in lifetime risk of coronary artery disease, a 2-fold increase in the risk of heart failure (HF), and significantly reduced survival compared to the general population without T2DM. In the past 15 years, assessment of CV risk with drug therapies for T2DM has become increasingly important, especially since the meta-analysis by Nissen and Wolski in 2007 found a significant increase in CV mortality with rosiglitazone, a thiazolidinedione (see Q&A video interview of Steven Nissen in this issue).^1^ This was followed by the United States (US) Food and Drug Administration (FDA) directing mandatory CV outcomes trials for all subsequent drugs approved for T2DM to assess any excess CV risk.^1^ Thereafter, dipeptidyl peptidase-4 inhibitors (DPP-4), glucagon-like peptide 1 receptor (GLP-1R) agonists, and sodium-glucose cotransporter-2 (SGLT2) inhibitors have been extensively evaluated for major adverse cardiovascular events (MACE) in patients at high CV risk independent of their glycemic effect. Herein, we provide a review of the CV risk profile of old and contemporary glucose-lowering therapies.

## Biguanides

Metformin belongs to the biguanides class of antihyperglycemic drugs and is the most commonly prescribed drug for T2DM owing to its low cost, multimodal mechanism of action, and low-to-moderate side-effect profile. It was first introduced in practice in 1957 and has since sustained its role in T2DM management. The United Kingdom Prospective Diabetes Study (UKPDS) group performed a major randomized controlled trial evaluating the effect of metformin and intensive antihyperglycemic therapy (insulin, glibenclamide, or chlorpropamide) compared with conventional glucose-lowering therapy in 5,102 patients.^2^ Patients receiving metformin had a significant reduction in all T2DM-related events compared with those receiving conventional therapy (relative risk reduction [RRR] 32%; hazard ratio [HR], 0.68; 95% confidence interval [CI], 0.53-0.87) and the intensive glycemic control group. Metformin was also found to significantly reduce all-cause mortality compared with conventional (RRR 36%; HR, 0.64; 95% CI, 0.45-0.91) and intensive glycemic control groups. There was a significant reduction in T2DM-related death (RRR 42%; HR, 0.58; 95% CI, 0.58-0.91) and myocardial infarction (RRR 39%; HR, 0.61; 95% CI, 0.41-0.89) with metformin compared with conventional therapy; however, no significant difference was observed compared with the intensive glycemic control group. This study, by far, was the most extensive evaluation of CV risks associated with metformin use. However, this trial was performed in the 1990s when therapies for T2DM were still limited, did not enrich trial participants at high CV risk, and did not have focused prespecified CV end points. Multiple meta-analyses suggested inconclusive evidence regarding the effect of metformin on CV outcomes in T2DM, with no significant reduction reported in end points of multiple adverse cardiac events, CV death, and all-cause mortality.^3,4^ However, a more recent meta-analysis of > 1 million patients with coronary artery disease reported a significant reduction in all-cause mortality in patients with baseline myocardial infarction (RRR 21%; HR, 0.79; 95% CI, 0.68-0.92) and HF (RRR 16%; HR, 0.84; 95% CI, 0.81-0.87) with metformin.^5^ The incidence of CV events in the overall cohort was also significantly reduced (RRR 17%; HR, 0.83; 95% CI, 0.73-0.89). The paucity of existing and ongoing trials makes it difficult to definitively ascertain the role of metformin in CV disease protection. There has been a keen interest in evaluating the efficacy of metformin in improving the CV profile in patients without T2DM. However, evidence from several trials has remained unclear with insufficient studies evaluating CV adverse events, including death.

## Thiazolidinediones

Thiazolidinediones, namely rosiglitazone and pioglitazone, were initially approved by the FDA in 1999 for use in T2DM. However, concerns were raised following a meta-analysis performed by Nissen and Wolski in 2007 evaluating CV outcomes with rosiglitazone therapy in T2DM.^6^ The occurrence of myocardial infarction was significantly increased (HR, 1.43; 95% CI, 1.03-1.98) with a borderline increase in CV death in patients on rosiglitazone therapy versus placebo (HR, 1.64; 95% CI, 0.98-2.74). The results were validated by an internal analysis by the FDA, and a black box warning was issued for rosiglitazone use in 2007. Soon after, an interim analysis of the RECORD (Rosiglitazone Evaluated for Cardiovascular Outcomes) trial,^7^ an unblinded study, reported a significant increase in the occurrence of incident HF in the rosiglitazone arm (1.7% vs 0.8%; HR, 2.24; 95% CI, 1.27-3.97); however, risk of major adverse CV events was otherwise not significantly different from placebo. The findings remained unchanged in the final analysis of the RECORD trial in 2009; however, the event rate for the primary end point was less than anticipated over the 5.5-year median follow-up period, resulting in the study being underpowered.^8^ These findings raised concerns about the CV efficacy of current glucose-lowering medications and those of future drug therapies, resulting in the FDA mandating an evaluation of CV outcomes through phase 4 clinical trials for antihyperglycemic drugs in 2008.^1^ Similar findings were not found with pioglitazone; in fact, it was found to decrease the composite risk of myocardial infarction and stroke in patients without, but at high risk for, T2DM who had recently suffered a stroke (9.0% vs 11.8%; HR, 0.76; 95% CI, 0.62-0.93) in the IRIS (Insulin Resistance Intervention After Stroke) trial.^9^ Currently, thiazolidinediones are not considered first-line therapies for T2DM management and are not recommended in HF patients or in those at high CV risk.

## Sulfonylureas

Sulfonylureas are one of the most popular glucose-lowering therapies due to their impressive glucose-lowering properties; however, given the potential CV risk associated with their use and the availability of newer therapies with a wide range of beneficial effects on CV and renal disease, their popularity has begun to dampen. Concerns regarding CV outcomes were first raised after the University Group Diabetes Program (UGDP) trial, involving 823 persons, revealed a significantly increased risk of CV death with tolbutamide compared to dietary restrictions alone.^10^ This led to the FDA issuing a black box warning of increased CV mortality with all sulfonylureas. These findings were in contrast to the UKPDS, which revealed a reduction in overall mortality associated with intensive glycemic control with sulfonylureas.^11^ Numerous meta-analyses have been performed to evaluate CV outcomes with sulfonylureas, with results largely neutral in some studies^12^ but more often indicating an increased CV risk.^13,14,15^ The CAROLINA (Cardiovascular Outcome Study of Linagliptin Versus Glimepiride In Patients with Type 2 Diabetes) trial is a recent study that compared the effect of glimepiride versus linagliptin on CV outcomes in 6,033 patients with T2DM at high risk of CV disease and a mean follow-up of 6.3 years^16^ Glimepiride was found to be non-inferior to linagliptin in reducing the occurrence of the MACE-3 (comprising CV death, nonfatal stroke, and nonfatal myocardial infarction) primary end point (12.0% vs 11.8%; HR, 0.98; 95% CI, 0.84-1.14). These findings do negate concerns regarding increased CV risk, at least for this specific sulfonylurea agent; however, large-scale contemporary randomized controlled trials of sulfonylureas similar to those performed for some of the newer drugs are still lacking.

## Insulin

Insulin is considered for the management of T2DM when oral therapies have failed to achieve optimal glycemic control and is considered the initial therapy of choice for patients with a high hemoglobin A1c (≥ 10%) at the time of diagnosis. Evidence for CV outcomes with insulin therapy is not encouraging. Apart from the findings described earlier with sulfonylureas, the VADT (Veterans Affairs Diabetes Feasibility Trial) study revealed that an intensive glucose-lowering approach was associated with a nonsignificant increase in CV events and no difference in mortality.^17^ The ACCORD (Effects of Intensive Glucose Lowering in Type 2 Diabetes) trial of 10,251 patients with established CV disease compared the effects of intensive glycemic control (77% on insulin) compared with standard therapy (55% on insulin) on CV outcomes.^18^ Intensive therapy with insulin with target hemoglobin A1c < 6% was found to worsen all-cause mortality compared with standard therapy (5.0% vs 4.0%; HR, 1.22; 95% CI, 1.01-1.46); however, a significant reduction in nonfatal myocardial infarction was observed with intensive therapy. The ORIGIN (Basal Insulin and Cardiovascular and Other Outcomes in Dysglycemia) trial was the only major study with an insulin-only treatment arm that evaluated CV outcomes with basal insulin therapy versus standard regimen in 12,537 patients with impaired glycemic control and at high risk for adverse CV events.^19^ No significant difference in the coprimary outcome of CV death, nonfatal myocardial infarction, nonfatal stroke, revascularization procedures, and HF hospitalizations was observed with insulin therapy compared with standard of care (28.6% vs 27.5%; HR, 1.04; 95% CI, 0.97-1.11). A meta-analysis of eight CV outcomes trials of contemporary drugs in patients receiving insulin as background therapy compared with those who were not on insulin revealed a higher risk of MACE-3 in patients on insulin therapy.^20^

## Glucagon-Like Peptide 1 Receptor Agonists

GLP-1R agonists act by promoting insulin activity, reducing gastric emptying, and inhibiting glucagon secretion, thereby improving glycemic control in T2DM. Exenatide was the first GLP-1R agonist approved for use in T2DM by the FDA in 2005, followed by several other drugs that were approved for routine clinical use. In 2019, the first oral form of this drug class (semaglutide) was approved for routine use after findings from the PIONEER 6 (Peptide Innovation for Early Diabetes Treatment 6) trial showed a significant reduction in levels of glycosylated hemoglobin and weight loss, with no significant increase in major adverse CV events.^21^

### Cardiovascular Outcome Trials

The ELIXA (The Evaluation of Lixisenatide in Acute Coronary Syndrome) trial was the first study that evaluated CV outcomes with a GLP-1R agonist (lixisenatide) in patients at high risk of CV events.^22^ The trial enrolled 6,068 patients who had experienced an acute coronary event within 180 days of enrollment with a median trial follow-up of 2 years. Lixisenatide was found to be noninferior but not superior to placebo in the occurrence of the primary composite outcome of CV death, nonfatal stroke, nonfatal myocardial infarction, and hospitalization for unstable angina (13.4% vs 13.2%; HR, 1.02; 95% CI, 0.89-1.17).

Subsequently, the LEADER (Liraglutide Effect and Action in Diabetes: Evaluation of Cardiovascular Outcome Results) trial evaluated CV outcomes with liraglutide in 9,340 patients at high risk of CV events (age > 50 years with at least 1 coexisting CV condition or age > 60 years with at least 1 risk factor for CV disease).^23^ The trial had considerably sicker patients compared to the ELIXA trial: approximately 81% of patients had established CV disease with a higher mean hemoglobin A1c level of 8.7% (vs 7.7% in ELIXA) and a longer median follow-up of 3.8 years. Liraglutide significantly reduced the occurrence of the MACE-3 primary end point compared to placebo (13.0% vs 14.9%; HR, 0.87; 95% CI, 0.78-0.97), mostly driven by a significant reduction in CV death (HR, 0.78; 95% CI, 0.66-0.93).

The SUSTAIN-6 (Trial to Evaluate Cardiovascular and Other Long-term Outcomes with Semaglutide in Subjects with Type 2 Diabetes) trial evaluated CV outcomes with injectable semaglutide in 3,297 patients at high risk for CV events—with a participant inclusion criteria and primary composite end point similar to that of the LEADER trial—for a median follow-up of 2.1 years.^24^ Injectable semaglutide was found to be noninferior and superior to placebo in significantly reducing the occurrence of MACE-3 (6.6% vs 8.9%; HR, 0.74; 95% CI, 0.58-0.95). The majority of CV benefit in the primary end point was derived from a significant reduction in nonfatal stroke (HR, 0.61; 95% CI, 0.38-0.99).

The EXSCEL (Exenatide Study of Cardiovascular Event Lowering) trial was the largest of this group of GLP-1R agonists studies, enrolling a total of 14,752 participants with a median follow-up of 3.2 years.^25^ The trial was designed to ensure that approximately 70% of enrollees would have a previous history of CV disease. Exenatide was found to be noninferior, but not superior, to placebo in reducing the occurrence of the MACE-3 primary end point (11.4% vs 12.2; HR, 0.91 [0.83-1.00]).

The HARMONY OUTCOMES (Effect of Albiglutide, When Added to Standard Blood Glucose Lowering Therapies, on Major Cardiovascular Events in Subjects With Type 2 Diabetes Mellitus) trial evaluated CV outcomes with albiglutide in 9,463 patients with established CV disease with a median follow-up of 1.6 years.^26^ Albiglutide significantly reduced the occurrence of the MACE-3 primary end point compared with placebo (7.0% vs 9.0%; HR, 0.78; 95% CI, 0.68-0.90), which was majorly driven by a significant reduction in myocardial infarction in the albiglutide arm (HR, 0.75; 95% CI, 0.62-0.91).

The REWIND (Dulaglutide and Cardiovascular Outcomes in Type 2 Diabetes) trial^27^ was designed to only evaluate the superiority of dulaglutide in reducing adverse CV outcomes in a low CV risk population, making it one of the first primary prevention studies for GLP-1R agonists. The trial enrolled 9,901 participants, with a history of existing CV disease in 31% of patients, but it had a longer median follow-up of 5.4 years. Dulaglutide was found to significantly reduce the occurrence of the MACE-3 primary end point compared with placebo (12.0% vs 13.4%; HR, 0.88; 95% CI, 0.79-0.99), predominantly attributed to a significant reduction in nonfatal stroke (2.7% vs 3.5%, *P* = .017).

The PIONEER 6 trial was unique because it evaluated CV outcomes with the administration of the first oral form of the GLP-1R class (semaglutide).^21^ The trial enrolled 3,183 patients at high risk of adverse CV events (approximately 84% of participants had either established CV disease or chronic kidney disease). The median follow-up was 15.9 months, the shortest among this group of trials, and hence resulted in a much lower overall event rate. Oral semaglutide was found to be noninferior but not superior to placebo in reducing the occurrence of the MACE-3 primary end point (3.8% vs 4.8%; HR, 0.79; 95% CI, 0.57-1.11).

The AMPLITUDE-O (Cardiovascular and Renal Outcomes with Efpeglenatide in Type 2 Diabetes) trial evaluated CV outcomes with efpeglenatide in 4,076 participants with established CV or kidney disease over a median follow-up duration of 1.8 years.^28^ Efpeglenatide was found to be superior to placebo in reducing the MACE-3 primary end point by 27% (7.0% vs 9.2%; HR, 0.73; 95% CI, 0.58-0.92). The treatment arm also had a significantly lower occurrence of HF hospitalization (1.5% vs 2.3%; HR, 0.61; 95% CI, 0.68-0.98).

**Figure 1:**
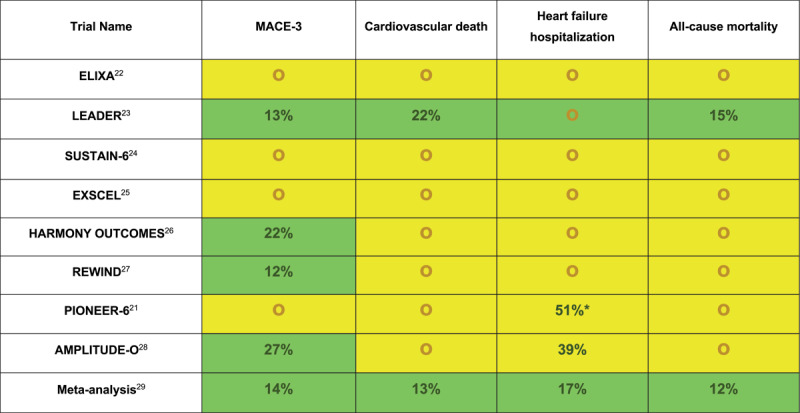
Cardiovascular outcomes in individual randomized controlled trials for glucagon-like peptide 1 receptor agonists. Relative risk reduction was only reported for statistically significant outcomes. ELIXA: The Evaluation of Lixisenatide in Acute Coronary Syndrome; LEADER: Liraglutide Effect and Action in Diabetes: Evaluation of Cardiovascular Outcome Results; SUSTAIN-6: Semaglutide and Cardiovascular Outcomes in Patients with Type 2 Diabetes; EXSCEL: Effects of Once-Weekly Exenatide on Cardiovascular Outcomes in Type 2 Diabetes; HARMONY OUTCOMES: Albiglutide and Cardiovascular Outcomes in Patients with Type 2 Diabetes and Cardiovascular Disease; REWIND: Dulaglutide and Cardiovascular Outcomes in Type 2 Diabetes; PIONEER 6: Oral Semaglutide and Cardiovascular Outcomes in Patients with Type 2 Diabetes; AMPLITUDE-O: Cardiovascular and Renal Outcomes with Efpeglenatide in Type 2 Diabetes; MACE-3: cardiovascular death, nonfatal myocardial infarction and nonfatal stroke; green highlight: statistically significant reduction in outcome; yellow highlight: no statistically significant difference in outcome or statistically significant noninferiority; red highlight: statistically significant increase in outcome * *P*-value not reported.

A meta-analysis of the above eight trials revealed a significant reduction in the MACE-3 end point compared to placebo by 14% (HR, 0.86; 95% CI, 0.79-0.94), which was for the most part driven by a reduction in CV mortality by 13% and nonfatal stroke by 16%.^29^ A significant 12% reduction in all-cause mortality and 10% reduction in HF hospitalizations were also reported. Overall, GLP-1R agonists have proven to significantly improve CV outcomes in patients with T2DM (Figure 1). The signal for reduction in HF hospitalizations has prompted several ongoing trials, including STEP-HFpEF-DM (Research Study to Investigate How Well Semaglutide Works in People Living With Heart Failure, Obesity and Type 2 Diabetes)^30^ and STEP-HFpEF (Research Study to Investigate How Well Semaglutide Works in People Living With Heart Failure and Obesity),^31^ evaluating the efficacy of injectable semaglutide in augmenting weight loss and HF quality of life measures.

## Dipeptidyl Peptidase 4 Inhibitors

**Figure 2:**
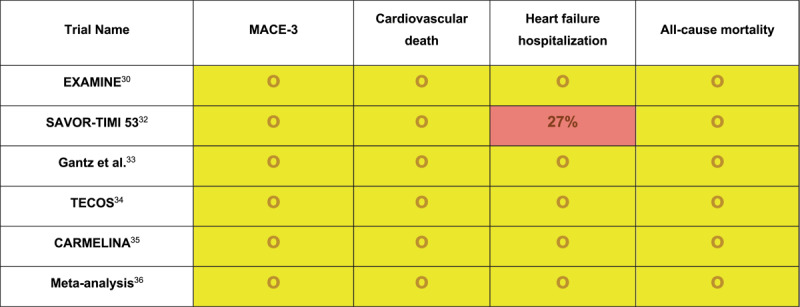
Cardiovascular outcomes in individual randomized controlled trials for dipeptidyl peptidase 4 inhibitors. Relative risk reduction or relative harm was only reported for outcomes that were statistically significant. EXAMINE: Alogliptin after Acute Coronary Syndrome in Patients with Type 2 Diabetes; SAVOR-TIMI 53: Saxagliptin and Cardiovascular Outcomes in Patients with Type 2 Diabetes Mellitus; TECOS: Effect of Sitagliptin on Cardiovascular Outcomes in Type 2 Diabetes; CARMELINA: Effect of Linagliptin vs Placebo on Major Cardiovascular Events in Adults with Type 2 Diabetes and High Cardiovascular and Renal Risk; MACE-3: cardiovascular death, nonfatal myocardial infarction, and nonfatal stroke; green highlight: statistically significant reduction in the outcome; yellow highlight: no statistically significant difference in outcome or statistically significant noninferiority; red highlight: statistically significant increase in outcome

DPP-4 inhibitors inhibit the enzyme dipeptidyl peptidase 4, which is responsible for the inactivation of GLP-1. This results in increased activity of GLP-1, which possesses a diverse set of antihyperglycemic properties as discussed earlier, making it an effective oral therapeutic option for T2DM. Sitagliptin was the first drug of this class that was approved by the FDA in 2006, with several others that have since been added to the armory. Like GLP-1R agonists, there have been several trials that have assessed CV outcomes with this drug class. However, unlike GLP-1R agonists, results have been rather mixed (Figure 2).

### Cardiovascular Outcome Trials

The EXAMINE (Examination of Cardiovascular Outcomes with Alogliptin versus Standard of Care) trial evaluated CV outcomes with alogliptin in patients at high risk of adverse CV events.^32^ The trial included a total of 5,380 patients who had experienced an acute coronary event within the previous 15 to 90 days, and it had a median follow-up of 18 months. Alogliptin was found to be noninferior to placebo in reducing the MACE-3 primary end point (11.3% vs 11.8%; HR, 0.96; upper limit of 95% CI < 1.16). However, there was a nonsignificant increase in HF hospitalizations reported in the alogliptin arm (3.9% vs 3.3%; HR, 1.19; 95% CI, 0.90-1.58). The latter finding was further evaluated in a post-hoc analysis of the trial, where the occurrence of HF hospitalizations as a first event was not significantly different between the alogliptin and placebo groups (3.1% vs 2.9%; HR, 1.07; 95% CI, 0.79-1.46).^33^

The SAVOR-TIMI 53 (The Saxagliptin Assessment of Vascular Outcomes Recorded in Patients with Diabetes Mellitus–Thrombolysis in Myocardial Infarction 53) trial had a much larger participant pool of 16,492 participants with an established history or risk of CV events and a longer median follow-up of 2.1 years.^34^ Saxagliptin was found to be noninferior to placebo in reducing the MACE-3 primary end point (7.3% vs 7.2%; HR, 0.89; 95% CI, 0.89-1.12). However, there was a significant increase in the number of HF hospitalizations in the saxagliptin arm compared with placebo (3.5% vs 2.8%; HR, 1.27; 95% CI, 1.07-1.57). The findings of increased risk of HF hospitalizations in both these trials led to the FDA issuing a safety warning for the use of alogliptin and saxagliptin in patients with T2DM and HF.

A randomized controlled trial for omarigliptin, a once-weekly DPP-4 inhibitor, was performed to assess CV outcomes in 4,202 patients with T2DM and established CV disease over a median follow-up of 1.8 years.^35^ No significant difference was observed between omarigliptin and placebo in the occurrence of the MACE-3 primary end point (5.5% vs 5.4%; HR, 1.00; 95% CI, 0.77-1.29). Omarigliptin also did not significantly reduce all-cause mortality and HF hospitalizations.

The TECOS (Trial Evaluating Cardiovascular Outcomes with Sitagliptin) trial evaluated CV outcomes with sitagliptin in 14,671 participants with established CV risk and a median follow-up of 3 years.^36^ Sitagliptin was found to be noninferior to placebo for the primary outcome of MACE-3 and hospitalizations for unstable angina (11.2% vs 11.4%; HR, 0.98; 95% CI, 0.88-1.09). There was also no significant difference in the rates of HF hospitalizations between the two arms (HR, 1.00; 95% CI, 0.83-1.20).

More recently, the CARMELINA (Cardiovascular and Renal Microvascular Outcome Study With Linagliptin in Patients With Type 2 Diabetes Mellitus) trial evaluated CV outcomes with linagliptin in 6,979 patients with a previous history of CV events or significant proteinuria and a median follow-up of 2.2 years.^37^ Linagliptin was found to be noninferior to placebo for the MACE-3 primary end point (12.4% vs 12.1%; HR, 1.02; 95% CI, 0.89-1.17). The rate of HF hospitalizations was also similar in both groups (6.0% vs 6.5%; HR, 0.90; 95% CI, 0.74-1.08).

A meta-analysis of these trials reaffirmed that DPP-4 inhibitors do not improve or worsen CV outcomes in T2DM, with no significantly increased risk of HF hospitalizations.^38^ Although later analyses have not revealed a significant association between DPP-4 inhibitor use and HF hospitalizations, major guidelines currently do not recommend saxagliptin and alogliptin for the management of T2DM in patients with HF.

## Sodium-Glucose Cotransporter 2 Inhibitors

**Figure 3:**
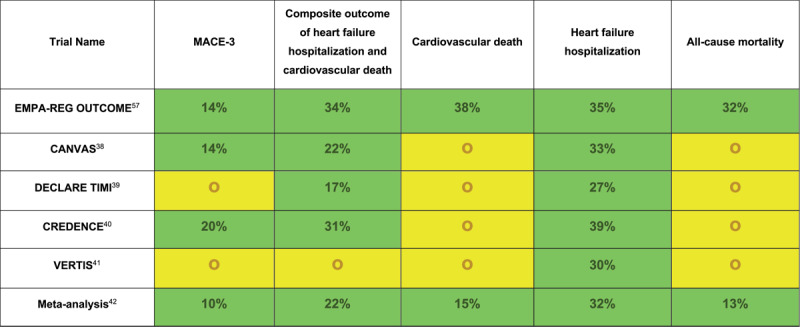
Cardiovascular outcomes in individual randomized controlled trials for sodium-glucose cotransporter-2 inhibitors. Relative risk reduction was only reported for outcomes that were statistically significant. EMPA-REG OUTCOME: Empagliflozin Cardiovascular Outcome Event Trial in Type 2 Diabetes Mellitus Patients; CANVAS: Canagliflozin and Cardiovascular and Renal Events in Type 2 Diabetes; DECLARE TIMI: Dapagliflozin and Cardiovascular Outcomes in Type 2 Diabetes; CREDENCE: Canagliflozin and Renal Outcomes in Type 2 Diabetes and Nephropathy; VERTIS: Cardiovascular Outcomes with Ertugliflozin in Type 2 Diabetes; MACE-3: cardiovascular death, nonfatal myocardial infarction, and nonfatal stroke; green highlight: statistically significant reduction in the outcome; yellow highlight: no statistically significant difference in outcome or statistically significant noninferiority; red highlight: statistically significant increase in outcome.

SGLT2 inhibitors have perhaps garnered the most attention in recent years. The mechanism of action for glycemic control is attributed to the inhibition of selective SGLT2 receptors located in the proximal convoluted tubules, inhibiting glucose resorption with resultant glucosuria. Canagliflozin was the first drug approved by the FDA for T2DM in 2013, followed by several others, all of which have undergone rigorous clinical trials to assess CV safety with favorable results (Figure 3).

### Cardiovascular Outcome Trials in Type 2 Diabetes Mellitus

The EMPA-REG OUTCOME (Empagliflozin Cardiovascular Outcome Event Trial in Type 2 Diabetes Mellitus Patients) trial was the first study that evaluated CV outcomes with an SGLT2 inhibitor in patients at high risk for CV events.^39^ The study enrolled 7,020 participants with established CV disease who were followed for 3.1 years. Remarkably, empagliflozin was not only found to be superior to placebo in reducing the MACE-3 primary end point by 14% (12.1% vs 10.5%; HR, 0.86; 95% CI, 0.74-0.99), primarily attributed to a significant reduction in CV death, but it also significantly reduced all-cause mortality with an RRR of 32% (5.7% vs 8.3%; HR, 0.68; 95% CI, 0.57-0.82). Moreover, a significant reduction in HF hospitalizations was also observed in the empagliflozin arm (2.7% vs 4.3%; HR, 0.65; 95% CI, 0.50-0.85). The results of this landmark trial set the stage for SGLT2 inhibitors to be comprehensively evaluated for their CV effects irrespective of their antihyperglycemic effect.

The CANVAS (Canagliflozin Cardiovascular Assessment Study) trial evaluated the CV profile of canagliflozin in 10,142 patients with established atherosclerotic CV disease or those with risk factors for CV disease. Canagliflozin was found to be superior to placebo in reducing the MACE-3 primary end point (26.9 events per 1,000 person-years [PY] vs 31.5 events per 1,000 PY; HR, 0.86; 95% CI, 0.75-0.97).^40^ HF hospitalizations were also significantly reduced with empagliflozin (16.3 per 1,000 PY vs 20.8 per 1,000 PY; HR, 0.78; 95% CI, 0.67-0.91).

The DECLARE TIMI 58 (Dapagliflozin Effect on Cardiovascular Events–Thrombolysis in Myocardial Infarction 58) trial was the largest SGLT2 inhibitor CV outcome study, enrolling a total of 17,160 participants with established CV disease over the longest median follow-up of 4.2 years.^41^ In this trial, dapagliflozin was found to be noninferior but not superior to placebo in reducing the MACE-3 primary end point (8.8% vs 9.4%; HR, 0.93; 95% CI, 0.84-1.03). However, the composite outcome of CV death and HF hospitalization was significantly reduced with dapagliflozin (4.9% vs 5.8%; HR, 0.83; 95% CI, 0.73-0.95), mostly driven by a significant reduction in HF hospitalizations (HR, 0.73; 95% CI, 0.61-0.88).

The CREDENCE (Evaluation of the Effects of Canagliflozin on Renal and Cardiovascular Outcomes in Participants With Diabetic Nephropathy) trial primarily evaluated renal outcomes associated with the use of canagliflozin in patients with T2DM.^42^ The trial enrolled 4,401 patients with no specific criteria for existing CV risk with a median follow-up of 2.6 years. The secondary composite outcome of CV death and HF hospitalizations was found to be significantly reduced in the canagliflozin arm (8.1% vs 11.5%; HR, 0.69; 95% CI, 0.57-0.83), with a significant individual reduction in both CV death (HR, 0.78; 95% CI, 0.61-1.00) and HF hospitalizations (HR, 0.61; 95% CI, 0.47-0.80).

The VERTIS CV (Cardiovascular Outcomes Following Ertugliflozin Treatment in Type 2 Diabetes Mellitus Participants With Vascular Disease) trial evaluated CV outcomes with ertugliflozin in 8,246 patients with established CV disease over a median follow-up of 3.5 years.^43^ Ertugliflozin was found to be noninferior but not superior to placebo in reducing the MACE-3 primary end point (11.9% in both groups; HR, 0.97; 95% CI, 0.85-1.11). However, a significant reduction in HF hospitalizations was reported in the ertugliflozin arm (2.5% vs 3.6%; HR, 0.70; 95% CI, 0.54-0.90).

The findings from these trials were pooled in a recent meta-analysis, which reported a comprehensive reduction in MACE-3, CV death, HF hospitalizations, and all-cause mortality in patients with T2DM at high CV risk.^44^ The consistent reduction in CV death and HF hospitalizations, in particular, across all major trials led the scientific community to believe that this drug class may have an independent effect on CV risk in the absence of T2DM. The reduction in HF hospitalizations across the spectrum of left ventricular ejection fraction (LVEF), in particular, suggested the utility of SGLT2 inhibitors in HF management.

### Heart Failure With Reduced Ejection Fraction

The DAPA-HF (Dapagliflozin and Prevention of Adverse Outcomes in Heart Failure) trial was the first study that evaluated the efficacy of an SGLT2 inhibitor in patients with HF with reduced ejection fraction (HFrEF) irrespective of T2DM.^45^ The trial enrolled 4,744 patients with stable, chronic HF with LVEF ≤ 40% who were followed over a period of 18 months. The results were groundbreaking: dapagliflozin was found to significantly reduce the primary composite outcome of CV death, HF hospitalizations, and urgent HF visits compared with placebo (16.3% vs 21.2%; HR, 0.74; 95% CI, 0.65-0.85). Moreover, all components of the primary composite outcome were individually found to be significantly reduced with dapagliflozin therapy. This study was followed by the EMPEROR-Reduced (Empagliflozin Outcome Trial in Patients With Chronic Heart Failure With Reduced Ejection Fraction) trial that evaluated the efficacy of empagliflozin in HF with LVEF ≤ 40%.^46^ The trial enrolled 3,730 patients with a median follow-up of 16 months. Empagliflozin significantly reduced the composite outcome of CV death and HF hospitalizations compared to placebo, with an RRR of 21% (19.4% vs 24.7%; HR, 0.75; 95% CI, 0.65-0.86). The results were driven by a 28% reduction in HF hospitalizations (HR, 0.92; 95% CI, 0.65-0.86). In a further pooled analysis from both these trials, a 13% reduction in all-cause mortality was observed (HR, 0.87; 95% CI, 0.77-0.98). Moreover, a significant 26% reduction in the composite end point of CV death and HF hospitalizations was observed with SGLT2 inhibitors (HR, 0.74; 95% CI, 0.68-0.82) with a 14% reduction in CV death (HR, 0.86; 95% CI, 0.76-0.98).

### Heart Failure With Preserved Ejection Fraction

The EMPEROR-Preserved (Empagliflozin Outcome Trial in Patients With Chronic Heart Failure With Preserved Ejection Fraction) trial was the first study that evaluated the efficacy of an SGLT2 inhibitor in patients with HF with mildly reduced (HFmrEF) and HF with preserved ejection fraction (HFpEF).^47^ The trial enrolled a total of 5,988 patients with HF and LVEF > 40% with NYHA II and NYHA III symptoms. The results were similar to those reported in EMPEROR-Reduced: empagliflozin reduced the primary composite outcome of CV death and HF hospitalizations by 19% (13.8% vs 17.1%; HR, 0.79; 95% CI, 0.69-0.90), driven by a significant 27% risk reduction in HF hospitalizations. The DELIVER (Dapagliflozin Evaluation to Improve the Lives of Patients With Preserved Ejection Fraction Heart Failure)^48^ trial evaluated the efficacy of dapagliflozin in patients HF with LVEF > 40%. The trial was similar in design to EMPEROR-Preserved except that the inclusion criteria were broadened to include patients with improved LVEF. The primary composite end point was comprised of unplanned HF hospitalization, urgent visits for HF, and CV death. A total of 6,263 patients were enrolled with a median duration of follow-up of 2.3 years. Dapagliflozin was found to significantly reduce the primary composite end point by 18% compared to placebo (16.4% vs 19.5%, HR 0.82; 95% CI, 0.73-0.92), driven largely by a significant 21% reduction in HF hospitalizations and urgent visits for HF (11.8% vs 14.5, HR 0.79; 95% CI, 0.69-0.91).

### Worsening Heart Failure

Findings from major trials in chronic stable HF prompted interest in the efficacy of empagliflozin in acute decompensated HF. The SOLOIST-WHF (Effect of Sotagliflozin on Cardiovascular Events in Patients With Type 2 Diabetes Post Worsening Heart Failure) trial evaluated the effect of early initiation of sotagliflozin following an episode of decompensated HF on CV outcomes in patients with T2DM.^49^ Unlike other selective SGLT2 inhibitors routinely used in HF and T2DM, sotagliflozin is a nonselective SGLT1 receptor (predominantly found in the gastrointestinal tract) and SGLT2 receptor antagonist. Although the trial ended early with a median follow-up for both study arms of approximately 9 months, a significant reduction in the primary composite end point of HF hospitalizations, CV death, and urgent HF visits was reported in the treatment arm (51.0 vs 76.3; HR, 0.67; 95% CI, 0.52-0.85). This effect was observed across the complete spectrum of LVEF, with statistical significance in the primary end point observed in both LVEF ≥ 50% and LVEF < 50%. Furthermore, the EMPULSE (A Study to Test the Effect of Empagliflozin in Patients Who Are in Hospital for Acute Heart Failure) trial evaluated the effect of empagliflozin initiation following an episode of decompensated HF across the LVEF spectrum irrespective of the presence of T2DM.^50^ The study of 530 participants reported a significant clinical benefit, defined as a hierarchical composite of death from any cause, the number of HF events and time to first HF event, or a 5-point or greater difference in change from baseline in the Kansas City Cardiomyopathy Questionnaire Total Symptom Score of in-hospital initiation of empagliflozin after 90 days of therapy (win ratio: 1.35; 95% CI, 1.09-1.68). The DICTATE-AHF (Efficacy and Safety of Dapagliflozin in Acute Heart Failure) trial is another ongoing trial that is evaluating the impact of in-hospital initiation of dapagliflozin in patients hospitalized for acute decompensated HF.^51^

### Myocardial Infarction

There is further evidence from preclinical studies to suggest that SGLT2 inhibitors may lessen pathological changes in the myocardium following an ischemic event, leading to decreased pathological biomarkers and infarct size, which may help prevent cardiac remodeling. The EMPACT-MI (A Study to Test Whether Empagliflozin Can Lower the Risk of Heart Failure and Death in People Who Had a Myocardial Infarction)^52^ and DAPA-MI (Dapagliflozin Effects on Cardiovascular Events in Patients With an Acute Heart Attack)^53^ trials are ongoing trials that are evaluating the primary end point of HF hospitalizations and all-cause mortality with SGLT2 inhibitor use after myocardial infarction.

## Guideline Recommendations for GLP-1R Agonists and SGLT2 Inhibitors

**Figure 4:**
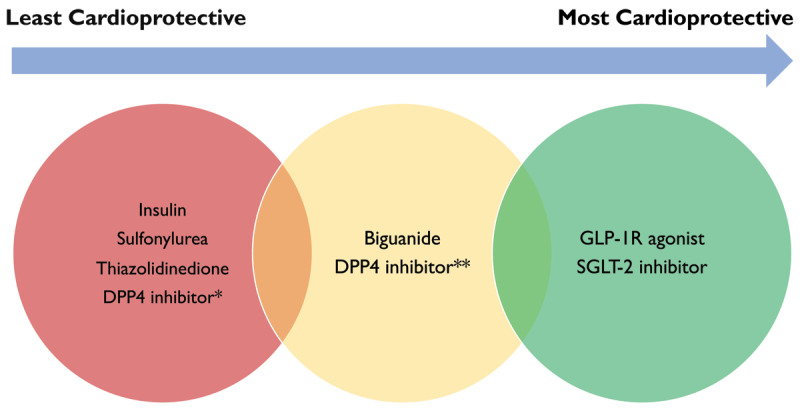
A general illustration of antihyperglycemic drugs and their comparative effectiveness of cardiovascular protection. * saxagliptin, alogliptin. ** sitagliptin, omarigliptin, linagliptin.

The American Diabetes Association recommends adding SGLT2 inhibitors and GLP-1R agonists to background glucose-lowering therapies in patients with atherosclerotic CV disease, HF, and/or chronic kidney disease.^54^ The American Heart Association/American College of Cardiology/Heart Failure Society of America (AHA/ACC/HFSA) 2022 Guideline Recommendations for Heart Failure and European Society of Cardiology 2021 Heart Failure Guidelines have assigned a class 1A recommendation for use of SGLT2 inhibitors (empagliflozin and dapagliflozin) in HFrEF, whereas the AHA/ACC/HFSA additionally denoted a Class 2A recommendation for use of SGLT2 inhibitor (empagliflozin) in patients with HFmrEF and HFpEF.^55^ Figure 4 illustrates a general outline of where each antihyperglycemic drug lies on the spectrum of CV protection.

## Effects of Glucocentric Drugs Beyond Glycemic Control

SGLT2 inhibitors reduce hemoglobin A1c by a modest 0.5% to 0.8% compared to approximately 1% with biguanides and 1.5% to 2.0% with sulfonylureas. However, the effect of SGLT2 inhibitors transcends beyond mere glycemic control, with a cardioprotective effect in patients with and without T2DM. The mechanism of action of SGLT2 inhibitors responsible for cardioprotection is still unknown; they do have a diverse array of metabolic targets apart from their glucosuric effect that likely plays a role in cardioprotection. GLP-1R agonists have a more pronounced effect on hemoglobin A1c (1% to 2%) similar to other older potent antihyperglycemic, but they offer additive CV protection due to a broader range of biomolecular effects. GLP-1R agonists are also potent weight-reducing agents and have a role in the pharmacological treatment of obesity irrespective of T2DM. Although a high hemoglobin A1c correlates with worse CV outcomes, the pathophysiology of CV disease in T2DM is multifaceted and is related to alterations in lipid/lipoprotein metabolism and sympathetic activity in addition to dysglycemia. The versatility of these contemporary drug therapies is driving the transition from a glucocentric to a more multisystem, cardiometabolic approach to T2DM management in patients at high CV risk.

## Future Directions

Several standardized, strictly regulated, industry-sponsored CV outcome trials have generated plenty of evidence for CV effects of newer antihyperglycemic agents. However, there is a lack of similar trials for older antihyperglycemic drugs (eg, biguanides, sulfonylurea, insulin) since they were already part of standard therapy for T2DM when the FDA mandate was introduced in 2008. Data is derived mainly from observational studies and a few trials performed several decades ago, in which the treatment arm was comprised of an intensive glycemic control group with multiple medications. The CAROLINA trial is a more contemporary study that highlighted the similarity in the efficacy of sulfonylureas compared with DPP-4 inhibitors in patients at high CV risk, indicating that sulfonylureas may not be as detrimental as once believed.^16^ There is a scarcity of high-quality CV outcome data in T2DM for metformin, which is the most commonly used first-line therapy; however, evidence for use in patients with established CV disease in the absence of T2DM has so far been equivocal. Hence, focused trials for ascertaining CV outcomes with these generic glucose-lowering therapies may be helpful in further optimizing medical therapy based on a patient’s CV risk and may potentially discover intrinsic cardioprotective effects of drugs irrespective of their glycemic effects, as in the case of SGLT2 inhibitors.

Given the efficacy of GLP-1R agonism and SGLT2 inhibition in CV prevention, there is a growing need for prospective outcome studies assessing the efficacy of simultaneous or sequential addition of both these drugs to T2DM management. The DURATION-8 (Exenatide Once Weekly Plus Dapagliflozin Once Daily Versus Exenatide or Dapagliflozin Alone in Patients with T2DM Inadequately Controlled With Metformin Monotherapy) and AWARD-10 (Dulaglutide as Add-on Therapy to SGLT2 Inhibitors in Patients With Inadequately Controlled Type 2 Diabetes) trials have already concluded that this dual therapeutic combination is safe and equally efficacious for glycemic control, with no significant difference in adverse events noted between dual therapy and individual drug therapy.^56^ There is evidence to support an additive effect on blood pressure, weight loss, lipid profile, and CV risk with dual therapy based largely on retrospective evaluation of trial data;^57^ however, simultaneous antagonism of multiple pathological pathways with these drugs may have a synergistic effect on CV outcomes in T2DM that require assessment with prospective randomized controlled trials.

New upcoming therapies include sotagliflozin, which is a dual SGLT-1 and SGLT2 inhibitor. SGLT-1 receptors are responsible for intestinal absorption of glucose; antagonism of these receptors results in decreased post-prandial glucose and improved glycemic control. The SCORED (Effect of Sotagliflozin on Cardiovascular and Renal Events in Patients With Type 2 Diabetes and Moderate Renal Impairment Who Are at Cardiovascular Risk) trial evaluated the efficacy of sotagliflozin in 10,584 subjects in patients at high CV risk over a median follow-up of 16 months.^58^ Sotagliflozin significantly reduced the composite end point of HF hospitalizations, CV death, and urgent HF visits (7.6% vs 10.0%; HR, 0.74; 95% CI, 0.63-0.88). The reduction was attributed to a significant reduction in HF hospitalizations and urgent HF visits (4.6% vs 6.8%; HR, 0.67 [0.55-0.82]). However, the risk of adverse events including diarrhea, volume depletion, diabetic ketoacidosis, and genital mycotic infections was significantly higher in the sotagliflozin group. Tirzepatide, a dual GLP-1R and glucose-dependent insulinotropic polypeptide (GIP) agonist, is also an emerging therapy that has demonstrated superior efficacy to injectable semaglutide in improving glycemic control in the SURPASS-2 (Tirzepatide versus Semaglutide Once Weekly in Patients With Type 2 Diabetes) trial.^59^ GIP agonism is a promising target as it enhances the lipid-lowering and fat deposition effects of GLP-1 and may have a synergistic effect in attenuating CV risk. Tirzepatide was recently found to significantly reduce body weight by up to 20% in patients with a body mass index of greater than 30 kg/m^2^ in the SURMOUNT-1 (Tirzepatide Once Weekly for the Treatment of Obesity) trial.^60^ It is also currently being evaluated for effects on CV outcomes in T2DM in the SURPASS-CVOT (A Study of Tirzepatide (LY3298176) Compared With Dulaglutide on Major Cardiovascular Events in Participants With Type 2 Diabetes) trial.^61^

## Conclusion

The fall of rosiglitazone has been a blessing in disguise as it led to mandatory CV outcome trials on newer therapies for T2DM. These trials revealed major CV benefits with GLP-1R agonists and SGLT2 inhibitors, which has led to a paradigm shift in the management of T2DM in patients with CV comorbidities and a recognition of the cardiometabolic effects of contemporary glucocentric drugs. Moreover, the cardioprotective effect of SGLT2 inhibitors extends to a reduction in CV mortality and in HF hospitalizations in HF across the LVEF spectrum, independent of its glucose-lowering effect, making the drug a mainstay in the management of HFrEF, HFmrEF, and HFpEF. Older glucocentric drugs are still commonly used by patients but have limited evidence for CV outcomes; hence, it is pivotal to augment T2DM regimens with GLP-1R agonists and SGLT2 inhibitors, especially in patients at increased CV risk.

## Key Points

- Contemporary glucose-lowering drugs have a diverse range of cardiometabolic effects that have led to a paradigm shift in the management of type 2 diabetes in patients at increased risk of adverse cardiovascular events.
- Dipeptidyl peptidase 4 inhibitors, glucagon-like peptide 1 receptor (GLP-1R) agonists, and sodium-glucose cotransporter-2 (SGLT2) inhibitors have been rigorously evaluated through cardiovascular outcomes trials in the management of type 2 diabetes.
- GLP-1R agonists and SGLT2 inhibitors were found to significantly reduce the risk of major cardiovascular events in diabetes mellitus and are recommended as first-line therapies for the management of type 2 diabetes in patients at increased cardiovascular risk.
- SGLT2 inhibitors have additionally been found to reduce heart failure hospitalizations and cardiovascular mortality in heart failure, irrespective of left ventricular ejection fraction or the presence of type 2 diabetes.
- There is a need for contemporary evaluation of cardiovascular outcomes with older-generation antihyperglycemic drugs, as most of the evidence for these medications is based on observational studies and is outdated.
- Prospective evaluation of dual therapy with GLP-1R agonists and SGLT2 inhibitors on cardiovascular outcomes is of great interest to assess if a synergistic effect exists with multimodal inhibition of pathological pathways in patients with and without type 2 diabetes.
